# Spatial and Temporal Patterns of Commercial Citrus Trees Affected by *Phyllosticta citricarpa* in Florida

**DOI:** 10.1038/s41598-017-01901-2

**Published:** 2017-05-09

**Authors:** Katherine E. Hendricks, Mary Christman, Pamela D. Roberts

**Affiliations:** 1 University of Florida, IFAS-Southwest Florida Research and Education Center, Department of Plant Pathology, Immokalee, FL 34142 USA; 2 University of Florida, Department of Statistics, Gainesville, Florida 32611 USA

## Abstract

Citrus black spot (CBS) caused by *Phyllosticta citricarpa*, is the most recent introduction of an exotic citrus pathogen into Florida and has been a challenge to control to date. Understanding the dispersal pattern of the disease within affected groves is vital in developing effective control strategies to limit the spread of the disease. The spatial pattern of CBS-affected trees was studied in two commercial ‘Valencia’ orange groves over three consecutive citrus seasons. Cluster analyses based on nearest-neighbor distance (F, G and J-functions) and pairwise distances between points (Ripley’s K function, Besag’s L function and the pair correlation function, *g*) were used to test the hypothesis of complete spatial randomness (CSR) of CBS infected trees within the groves. In both groves, the hypothesis of CSR was rejected for all tests performed including quadrats testing (2 × 2 trees up to 10 × 10 trees). The relationship between tree age and disease was assessed at one experimental site. Citrus trees bearing fruit for the first time accounted for approximately 13% of trees positive for disease and were located within areas of heavy disease pressure. These findings support short distance movement of inoculum as the main spread of disease in the groves studied.

## Introduction

*Phyllosticta citricarpa* the causal agent of citrus black spot (CBS) was first discovered in southwest Florida in April 2010^1^, Uganda in 2006^2^, Cuba in 2010^3^ and more recently in Ghana in 2012^4^. Under favorable conditions new infections can be initiated by either the sexual or asexual spore (ascospore or conidia, respectively). Ascospores are considered the major source of inoculum^5, 6^ and are released from pseudothecia typically formed in infected leaf litter following periods of wetting and drying. The sexual spores are released during wetting events, ejected into the air and dispersed into the canopy and beyond by wind, constituting long distance dispersal^7^. Conidia are the asexual spore produced in the leaf litter and on infected fruits, twigs and other infected citrus tissue. Conidia rely on splash dispersal to reach susceptible tissue, typically infecting susceptible tissue below its site of origin. Conidia produced in the leaf litter can only reach susceptible tissue through splash dispersal and hence has historically been considered to have a minor role in the disease cycle^6^. Once spores land on susceptible tissue, they penetrate the cuticle and epidermis to form a quiescent infection. Symptom expression is usually not seen in citrus leaves with the exception of lemons but occurs on the fruit as the fruit ripens.

In Florida however, mating type locus studies have not been able to find both mating types necessary for the production of ascospores^8^. This may indicate an atypical disease cycle in Florida. Based on this criterion it is believed that new infections are due to short range dispersal of the asexual spore, the conidia within the grove. Recent studies done in the laboratory suggest that conidia can be spread by splash and wind^9^ within tree-to-tree distances currently found in Florida groves.

Southwest Florida’s climate is divided into two distinct seasons based on rainfall pattern. The rainy season begins in June and extends to October accounting for approximately 70% of annual rainfall. The dry season runs from November through to May. Annual temperatures range from 15–18 °C to the high 20 s °C, with average temperatures between 23 and 24 °C, encompassing the optimum temperature for ascomata formation (21–28 °C)^7, 10^. In addition to the rainfall duality, Florida has a history of hurricanes and tropical storms. The latter is of much concern as conditions associated with these events have been associated with long-distance dissemination of citrus pathogens such as *Xanthomonas citri* subsp. *citri*, the causal agent of citrus canker^11–15^. It is possible that these events may play a significant role in future movement of *P*. *citricarpa* in the state of Florida.

The environmental conditions conducive for infection by *P*. *citricarpa* are temperatures between 24 and 30 °C^16^ and a minimum leaf wetness of 8 to 12 hours at optimal temperatures^9, 17^. Ascospore release occurs within a minimum of 20 min of leaf wetting^10^ and infection and colonization of susceptible tissue have been estimated at a minimum of 15 h in the presence of free standing water^18^. Our hypothesis was that the disease exhibits a clustered pattern in support of the conidia as the major source of inoculum within an infected grove in Florida rather than the ascospore.

## Results

### Weather Data and Susceptibility

Between January 1, 2010 and December 31, 2015 there were a total of 97, 44 and 139 days where ‘Valencia’ citrus fruit were susceptible to infection by the fungus and weather parameters were conducive to infection for IN1, IN2 and IN3 respectively (Table 1). Based on predicated flush of ‘Valencia’ sweet oranges in the study area, susceptible fruit were on the tree between April and August of each year. In 2011, 2012, 2013 and 2015, susceptible fruit were on the trees as early as the latter portion of February. In 2010, 2011, 2013, 2014 and 2015, susceptible fruit were expected to be on the tree into the early portions of September. Rainfall data for susceptible months is given in Table 2.

**Table 1:** Weather and Susceptibility Data associated with ‘Valencia’ orange groves surveyed in Florida for citrus black spot caused by *Phyllosticta citricarpa*.

Year	Susceptible Days^1^	Temperature (24–40 °C)	RH ≥ 90% for 8 hr	Rainfall ≥ 0.25 mm	IN1^2^ (days)	IN2^3^ (days)	IN3^4^ (days)
2010	173 (3/25–9/13)	157	76	64	32	66	55
2011	194 (2/23–9/4)	174	92	56	29	81	48
2012	198 (2/15–8/30)	176	107	61	36	96	52
2013	217 (2/6–9/10)	155	177	91	57	136	58
2014	194 (3/29–10/8)	169	128	89	52	107	70
2015	212 (2/17–9/16)	185	152	95	69	133	82

^1^Susceptible Days are the number of days when fruit are on the tree and are considered susceptible to infection by *Phyllosticta citricarpa* (between fruit set and 5 month post fruit set (mpfs)). Dates in brackets represent the estimated date of earliest fruit set and latest date of susceptibility at 5 mpfs.

^2^IN1 = RH ≥ 90% for 8 hr + Total daily rainfall ≥ 0.25 mm + Temperature (24–40 °C) + Susceptible Days.

^3^IN2 = RH ≥ 90% for 8 hr + Temperature (24–40 °C) + Susceptible Days.

^4^IN3 = Total daily rainfall ≥0.25 mm + Temperature (24–40 °C) + Susceptible Days.

**Table 2:** Cumulative Rainfall in mm for Susceptible Days^1^ from 2010 to 2015 measured at the FAWN, southwest Florida Immokalee weather station.

Year	2010	2011	2012	2013	2014	2015
Susceptible Days^1^	(3/25–9/13)	(2/23–9/4)	(2/15–8/30)	(2/6–9/10)	(3/29–10/8)	(2/17–9/16)
February	.	0	11.50	59.23	.	10.34
March	80.82	59.90	14.61	61.21	19.48	50.85
April	183.01	96.03	39.68	76.45	72.11	85.04
May	127.27	46.64	25.23	101.30	141.81	141.78
June	125.32	137.50	164.09	350.80	140.69	172.80
July	196.15	100.73	100.35	192.23	171.53	143.28
August	126.99	130.61	192.35	216.53	190.96	209.07
September	58.39	18.72	219.74	122.86	218.34	160.71
October	.	.	.	.	4.47	.
Cumulative rainfall (mm)	897.95	590.13	767.55	1180.61	959.39	973.87

^1^Susceptible Days are the number of days when fruit are on the tree and are considered susceptible to infection by *Phyllosticta citricarpa* (between fruit set and 5 month post fruit set (mpfs)). Dates in brackets represent the estimated date of earliest fruit set and latest date of susceptibility at 5 mpfs. A period (.) indicates that there was no days in the respective month where there were fruit on the tree, therefore no rainfall data is reported for that month.

### Spatial Pattern

**Figure 1:**
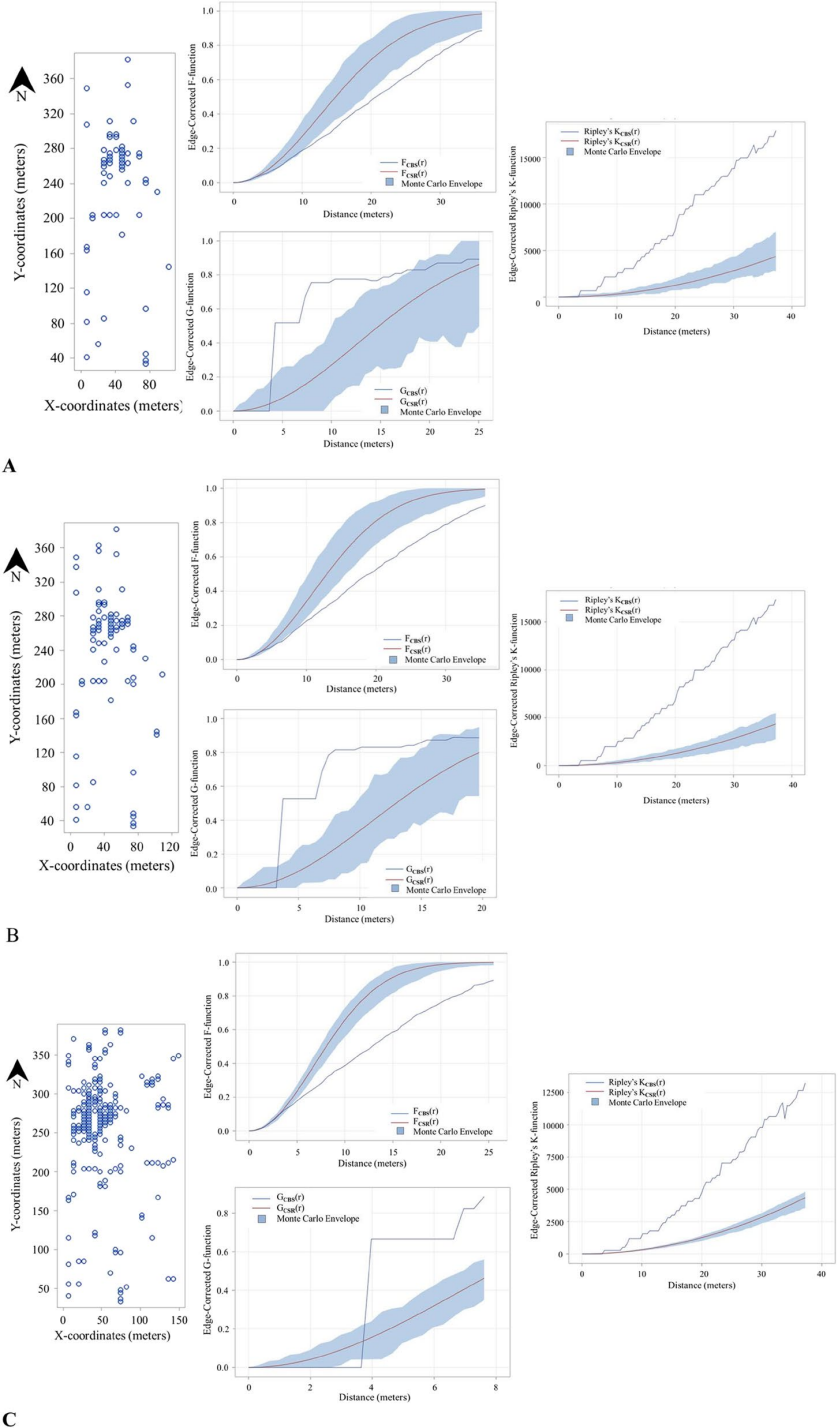
Grove I point pattern analyses. Comparisons of point pattern analyses for Grove I for three contiguous citrus seasons (Panel A) 2013–2014, (Panel B) 2014–2015 and (Panel C) 2015–2016. Each panel consists of a plot map of CBS positive trees (open circles; left), edge-corrected F- and G-functions (middle) and edge-corrected Ripley’s K-function (right). Positive trees contain at least one fruit with a single hard-spot lesion. Values of F_CBS_(r) < F_CSR_(r), and of G_CBS_(r) > G_CSR_(r) that falls outside of the Monte Carlo envelopes, indicate statistically significant clustering. For each graph, the red line corresponds to a Poisson process representing complete spatial randomness (CSR) and the blue shaded area is the Monte Carlo envelope that corresponds to the 5th to 95th percentiles of CSR, F(r), G(r) and Ripley’s K(r) respectively for values based on randomizations of the original data.

**Figure 2:**
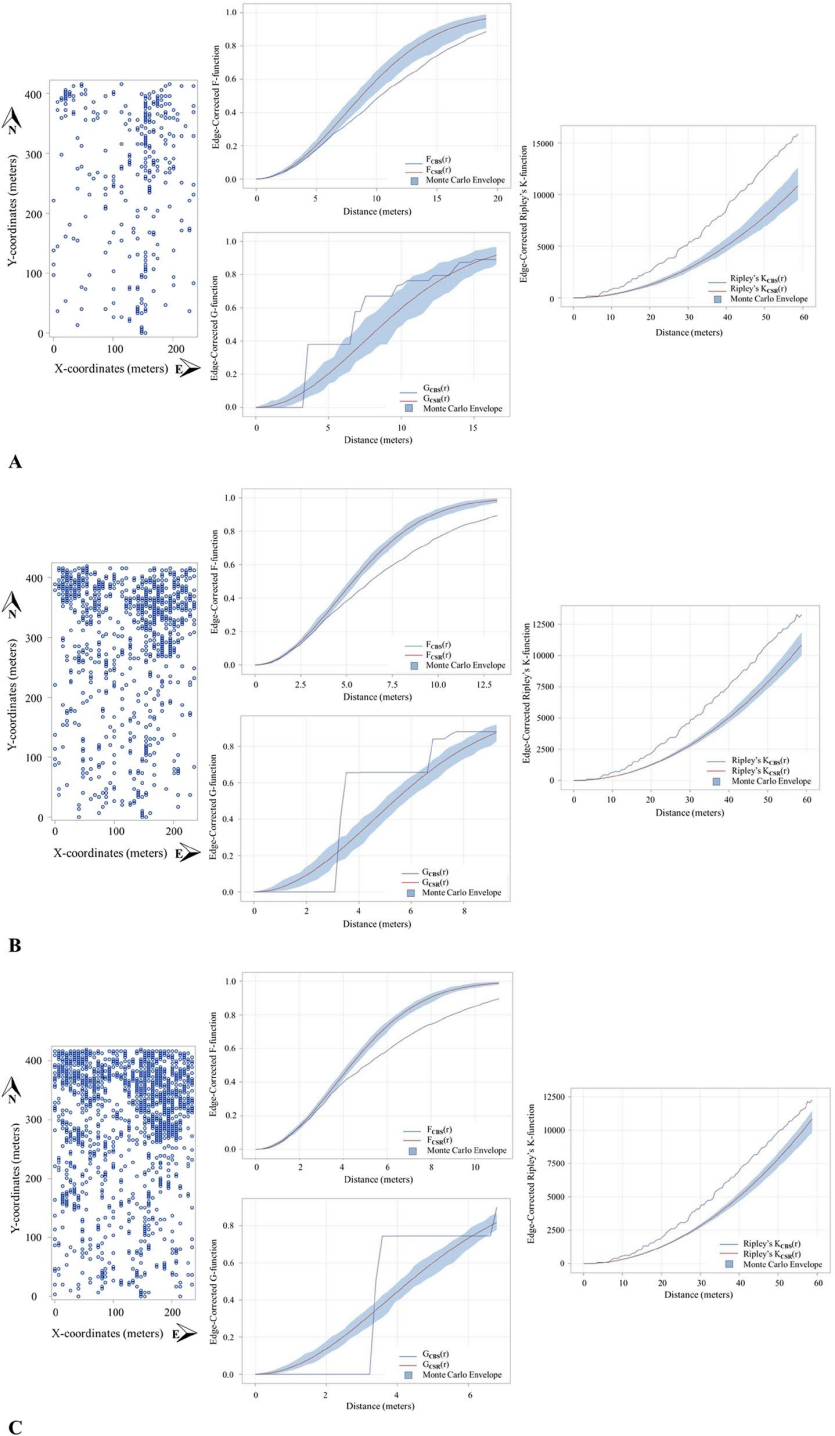
Grove II point pattern analyses. Comparisons of point pattern analyses for Groves II for three contiguous citrus seasons (Panel A) 2013–2014, (Panel B) 2014–2015 and (Panel C) 2015–2016. Each panel consists of a plot map of CBS positive trees (open circles; left), edge-corrected F- and G-functions (middle) and edge-corrected Ripley’s K-function (right). Positive trees contain at least one fruit with a single hard-spot lesion. Values of F_CBS_(r) < F_CSR_(r), and of G_CBS_(r) > G_CSR_(r) that falls outside of the Monte Carlo envelopes, indicate statistically significant clustering. For each graph, the red line corresponds to a Poisson process representing complete spatial randomness (CSR) and the blue shaded area is the Monte Carlo envelope that correspond to the 5th to 95th percentiles of CSR, F(r), G(r) and Ripley’s K(r) respectively for values based on randomizations of the original data.

In Grove I, the disease progressed eastward as reflected in the increase in the disease incidence and enlarging of the plots in an easterly direction as shown in Fig. 1A–C, from 100 meters (2013–2014 citrus season) to 150 meters (2015–2016 citrus season). Incidence increased from approximately 2.7% at the beginning of the study to 9.3% in the third year of study. Intensity increased with each season from 0.0010 (2013–2014) to 0.0013 trees per m^2^ (2014–2015) and finally 0.0034 trees per m^2^ during 2015–2016 citrus season. In Grove II, the disease incidence in the study area increased from 6.95% during the 2013–2014 citrus season to 26.57% in the 2015–2016 citrus season. Most of the infected fruit could be found in the northern region of the study area (Fig. 2A–C). The average intensity increased from 0.0029 to 0.012 CBS positive trees per m^2^ between the 2013–2014 and 2015–2016 citrus seasons. Quadrat analysis indicated clustering of diseased trees in both groves from the smallest quadrat size (2 trees × 2 trees) to the largest (10 trees × 10 trees; Table 3).

**Table 3:** Percentage of quadrats with at least one tree with citrus black spot (*P*), caused by *Phyllosticta citricarpa*, in groves I and II, and the respective binomial dispersion indexes^1^ (D) for different combinations of quadrat sizes for three years studied.

Quadrat size	Grove I							Grove II						
	No. quad	2013–2014		2014–2015		2015–2016		No. quad	2013–2014		2014–2015		2015–2016	
		P	D	P	D	P	D		P	D	P	D	P	D
2 by 2	561	7.31	1.6382*	9.63	1.7227*	21.39	1.7294*	1134	19.84	1.2340*	40.92	1.4205*	54.94	1.3669*
3 by 3	238	14.29	2.2436*	17.65	2.5087*	32.35	2.9809*	504	35.52	1.5956*	61.11	2.2228*	73.02	2.3075*
4 by 4	125	24.00	3.0243*	27.20	3.3065*	46.40	4.3696*	279	49.10	2.0279*	76.34	3.2798*	89.25	3.5444*
5 by 5	80	31.25	3.0982*	36.25	4.0599*	56.25	6.2928*	175	61.14	2.7838*	86.29	4.5436*	95.43	4.8904*
6 by 6	51	43.14	4.1389*	50.98	4.0352*	68.63	8.0235*	126	76.98	2.8944*	92.86	6.1345*	98.41	6.4293*
7 by 7	42	52.38	4.9178*	57.14	4.6585*	76.19	8.8599*	90	87.78	3.9261*	97.78	7.3530*	100	8.4054*
8 by 8	24	50.00	8.7071*	54.17	11.390*	79.17	18.190*	60	96.67	3.9723*	98.33	9.9384*	100	11.704*
9 by 9	22	54.55	8.6992*	59.09	10.439*	86.36	18.724*	56	98.21	4.2962*	100	10.546*	100	12.696*
10 by 10	20	60.00	7.5078*	65.00	9.2035*	90.00	20.099*	36	97.22	4.3480*	100	15.073*	100	18.513*

Pearson chi-square test for complete spatial randomness (CSR), values of p < 0.05 indicate a departure from CSR. Null hypothesis: There is no difference in the count of observed quadrats with at least one tree with fruit exhibiting CBS lesions when compared to the expected count of a homogeneous Poisson process. *p < 0.0001.

The three nearest-neighbor functions, F, G and J indicated that CBS positive trees were clustered in both Grove I and Grove II for all 3 years (Figs 1 and 2 respectively). Ripley’s K function, Besag’s L function and the pair correlation function also confirmed clustering of CBS positive trees. For Grove I and II, Ripley’s K indicated statistically significant clustering above 4.08 m and 6.5 m respectively regardless of year.

### Grove II: Tree Age and Citrus Black Spot

**Figure 3:**
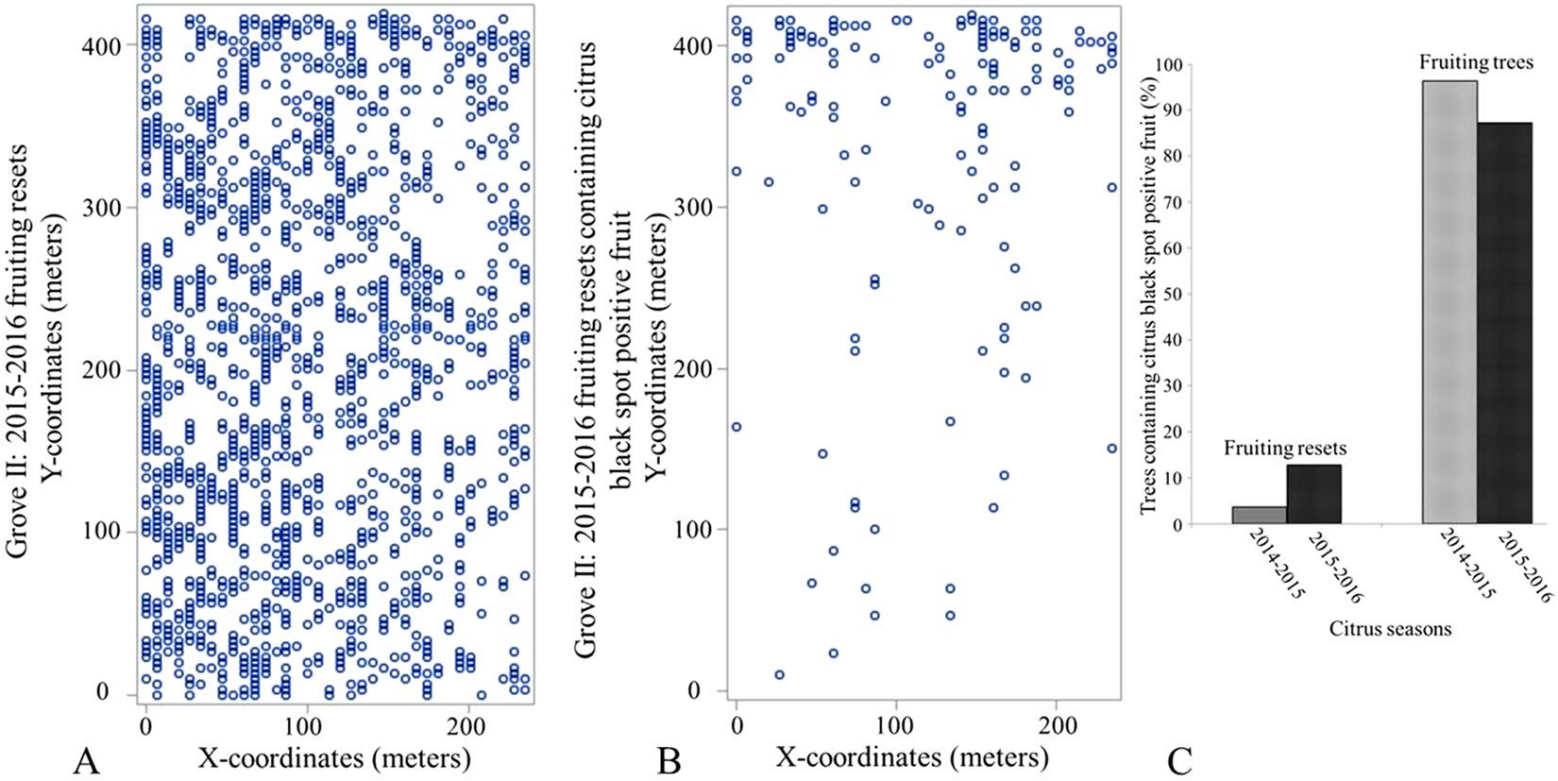
Grove II: Fruiting resets for the 2015–2016 citrus season. (**A**) – Spatial distribution of fruiting resets (open circles). Quadrat analysis of fruiting resets regardless of CBS status indicated random distribution of fruiting resets for 2 × 2 tree quadrats (dispersion index, D = 1.02; p-value = 0.347). For quadrat sizes 3 × 3 trees to 10 × 10 trees fruiting resets were statistically significantly clustered with dispersion indices, D ranging from 1.32 to 4.15; p < 0.001 for all quadrats. (**B**) – Spatial distribution of fruiting resets with fruit positive for citrus black spot (open circles). Quadrat analysis indicated disease clustering in fruiting resets with fruits positive for citrus black spot for all quadrat sizes tested (2 × 2 trees to 10 × 10 trees; D ranging from 1.25 to 11.03, p < 0.001 for all quadrat sizes). (**C**) – Column chart comparing the proportion of trees classified as fruiting resets and fruiting trees containing at least one fruit with a single hard-spot lesion in the 2014–2015 and 2015–2016 citrus season. Fruiting resets represent resets fruiting for the first time in their respective seasons.

In the 2014–2015 citrus season, there were 1499 trees classified as resets and of these 98% (1471) fruited in the following citrus season. One hundred and twenty-two (8.3%) of these newly fruiting resets contained fruit that was positive for CBS in the 2015–2016 citrus season. The vast majority of these positive fruiting resets were located within the northern portions of the study area where disease pressure was highest (Fig. 3).

During the 2015–2016 citrus season, trees were categorized as new resets, resets, fruiting resets and fruiting trees. There were 1153 trees with fruit positive for CBS, of these 148 were fruiting resets, accounting for approximately 13% of positives and 1005 were fruiting trees (87.2%). Fruiting resets made up 36.4% (1330/3656) of fruiting citrus within the study, while fruiting trees made up the remaining 63.6% of which 43.2% contained fruit positive for CBS. For trees with CBS positive fruit, there were significantly less resets fruiting for the first time (Category 3) in the 2015–2016 citrus season than fruiting trees (Category 4; 12.8% vs 87.2%; *χ* ^2^ p < 0.0001).

The average intensity for fruiting reset (Category 3) was 0.013475 per m^2^ in the 2015–2016 citrus season within the study area. Second-order characteristics, Ripley’s K, Besag’s L and G functions all indicate complete spatial randomness above 10 meters. For fruiting resets with CBS positive fruit, the average intensity was 0.001499 per m^2^ during the 2015–2016 citrus season. The nearest-neighbor, F, G and J functions all indicate significant clustering between 4 and 15 meters. While the second-order characteristics, Ripley’s K and Besag’s L functions indicate significant clustering between 4 and 35 meters.

### Sample Size

**Figure 4:**
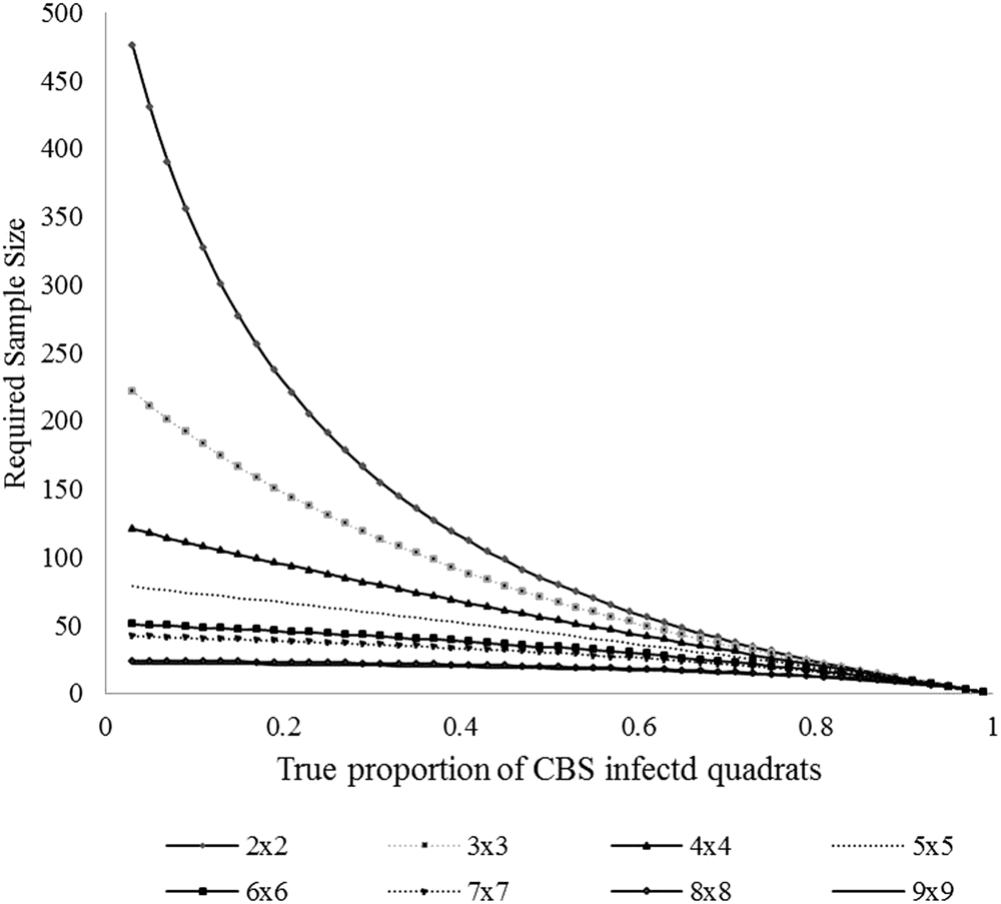
Required sample size for Grove I as a function of the true proportion of CBS infected quadrats for a (1 − α)100% = 95% confidence interval with 20% relative precision. Each line represents the required sample size for differing sizes of quadrats containing differing numbers of trees.

Using the observed annual proportions of infected trees as *p* in eq. (2), the minimum number of citrus trees needed to estimate disease incidence within each grove with 20% relative precision and 95% confidence was 1331, 1184 and 647 for Grove I for the 2013–2014, 2014–2015 and 2015–2016 citrus seasons respectively. For Grove II, minimum sample size estimates were 979, 413 and 251 for citrus seasons 2013–2014, 2014–2015 and 2015–2016 respectively. Using the observed annual proportion of infected quadrats as *p* (Table 3), the number of 2 by 2 quadrats required was 385, 347 and 217 for Grove I and 290, 124 and 74 from Grove II for citrus seasons 2013–2014, 2014–2015 and 2015–2016 respectively. The number of quadrats required to estimate the incidence of CBS with 95% confidence and within 20% of the true incidence in Grove I is shown for each quadrat size (2 by 2 to 10 by 10) in Fig. 4.

For the 2014–2015 citrus season, a total of 60,391,000 bearing citrus trees (orange, grapefruit and tangerine) were reported on a total of 456,000 acres in Florida, with an average of 132 trees per acre. At the lowest incidence rate surveyed of 2.7% (Grove I, 2013–2014 citrus season) there is a 95% chance that a positive tree will be observed in 83 or fewer surveyed trees within an average acre planting of 132 trees. At the highest incidence rate surveyed of 26.6% (Grove II, 2015–2016 citrus season) there is a 96% chance that a positive tree will be observed in 10 or fewer surveyed trees within an average acre planting of 132 trees. As the percent incidence of disease decreases the number of trees needed to be surveyed before finding a positive tree will increase.

## Discussion

The objective of this study was to determine the spatial distribution patterns within two CBS positive groves over time. The results indicate that the spatial pattern is non-random, i.e. evidence of CBS is clustered at all levels of analysis, similar to the situation found in Brazil^19^. A caveat to this study is the fact that symptom expression is required to positively identify a CBS infected tree. This does not mean that trees with no symptoms are negative as the organism has an extensive latent period^6, 20^.

This finding of clustering of the disease symptoms within affected groves may indicate that the spread of disease occurs over very short distances, which may be attributed to the asexual spores (conidia). Inoculum sources of *P*. *citricarpa* are the conidia and ascospore (the sexual spore). The conidia are produced on infected deadwood, twigs, leaves and fruits and are distributed by water (irrigation or rainfall). Once pycnidia mature, moisture events initiate the release of conidia as a mucilaginous mass from the osteole. These can then either be splash dispersed to nearby susceptible tissue or splash–wind driven. Splash dispersal of conidia from infected material is most likely the source of infection within the tree and between neighboring trees within rows in this study. Wind driven rain is likely the conveyor of conidia between rows (across swales and roadways, approximately 6.7 m or less). Recent findings suggests that rain combined with wind speeds of 7 m/s (25.2 km/h) has the potential of carrying aerosolized droplets across a distance of 8 m and to heights of 75 cm^9^. In addition, droplets ≤1 mm were likely to contain 0 to 4 conidia^9^. Together this may indicate in theory the potential for single conidia to travel greater distances that previously thought.

Field studies in Brazil indicate short distance dispersal of <0.8 m when symptomatic fruits and twigs were used as inoculum sources in a CBS–free experimental grove^21^. This distance is well below the average spacing in Grove II (6.70 m × 3.36 m) but within the canopy distances among trees (Category 4) where branches may overlap to form a continuous canopy. In this study where newly fruiting resets have been planted consecutively in rows (3.36 m spacing) and across rows (6.70 m spacing) well outside the 0.8 m of splash dispersed conidial influence, a large portion of these within the northern area of the study were found to have fruit with CBS symptoms. This along with published data suggesting that the sexual spore, which is thought to be responsible for long distance dissemination of the disease has not been found in Florida^8, 22^ suggest that the conidia is travelling greater distances than previously attributed to it. Whether this is on infected twigs and leaves from diseased trees or by wind driven rain/irrigation is not known. One could speculate that the sheer numbers of conidia present in the northern portion of the study area increases the likelihood of wind driven rain/irrigation containing conidial spores. It is more likely that under hurricane or tropic storm conditions, diseased twigs and branches (with or without fruit) may disseminate the disease over greater distances as seen with citrus canker^11–15^.

A combination of events is required for the spread of disease. For citrus black spot these are the presence of the disease causing agent – *Phyllosticta citricarpa*; the correct environmental conditions for infection and establishment of the disease; and, the presence of susceptible tissue – citrus (fruit, leaves and twigs). All three exist in Florida. Published data taken from other regions where citrus black spot is endemic have been used in lieu of local information, due to the lack thereof, to estimate environmental conditions conducive to infection. In southwest Florida between 2010 and 2015, suspect favorable environmental conditions for CBS infection occurred consistently between April and August of each year. In some years, these conditions appeared to start as early as February and extended until October. This was due to a combination of total daily rainfall ≥0.25 mm, temperatures between 24 and 40 °C for a minimum of 8 hr and the presence of susceptible fruit (fruit set to 5 months post fruit set) occurring within the same time frame and geographical location. These conditions increase the likelihood for conidia maturation and release, splash dispersal onto susceptible tissue and infection of said tissues. A caution to the strict definitions used here for assigning periods of total daily rainfall ≥0.25 mm is the consideration that extended periods of rainfall or heavy rainfall have the potential to wash inoculum out of the trees and away from susceptible tissues. Secondly, although the data for weather conditions are relevant to the Immokalee area, within grove weather data would have allowed the definitions for IN1 thru IN3 to be more accurately determined, as irrigation events and tree cover tends to increase and maintain high humidity for longer periods of time than in an open field. Even with these considerations, the environmental conditions above may have played a role in the increase incidence of disease seen over the three year study.

The incidence of CBS increased from 2.7% to 9.3% in Grove I and 7% to 26.6% in Grove II over the three seasons. This increase is not unique to Florida, as a doubling of disease incidence within a single season (10.7% to 22.7%) has been documented in ‘Valencia’ groves in Brazil^19^. And when considering ‘Valencia’ groves where there is an overlap of fruit between the current (for example the 2013–2014 crop) and the following season (2014–2015 crop) there is more likelihood of infected fruits contributing to the following season’s disease incidence. Using IN2 to define days when environmental conditions were conducive for infection, there were on average 125 days (range 107–136) where the humidity was high enough (>90% for 8 consecutive hours) to maintain standing water on leaves and fruits, perhaps even to runoff. Timmer and colleagues noted that, “*In Florida*, *leaf wetness durations exceed 3* *h nearly every night and are often 10 to 12* *h*”^23^. *Guignardia* spp, such as *G*. *psidii* which causes black spot symptoms on guava required as little as 6 hr of wetting for germination and appressorium formation^24^ and to establish disease^25^. Similarly conidia of *G*. *bidwellii*, which causes black rot of grapes, requires a minimum of 6 hr to cause infection on leaves at 26.5 °C and 9 hr at 29 °C^26^. It is plausible to speculate that the environmental conditions defined by IN1, IN2 and IN3 contributed to the increase in diseased incidence from one citrus season to the next.

A number of cultural practices have been suggested to reduce and/or control CBS. These include the removal of infected fruit to reduce inoculum load especially where there is an overlap of fruit as occurs in ‘Valencia’ groves. Harvest of ‘Valencia’ oranges took place between March and April of each year of study. Despite the removal of infected fruit during harvest, the incidence of CBS still increased over the three seasons. It is possible that a portion of these fruit were infected during the first 20 to 40 days post fruit set due to inoculum from ripening fruit. Additionally splash dispersal from other infected citrus tissues, such as dead twigs within the canopy^27^ may have also contributed to the increase in incidence of the disease over the three years of study as has been suggested by research in Brazil^21^. Failure to remove off season infected fruit, trimming and removal of dead tree limbs and twigs also contributes to sustaining inoculum sources in the grove.

Long distance dispersal of the disease has been attributed to the ascospore in South African, Asia and Australia. However, in Brazil whose micro-climates are closest to that of Florida, the conidia are considered the major source of disease spread^21^. Additionally, there is no evidence at this time that both mating types are present in Florida^8^ and multi-locus studies of *P*. *citricarpa* suggest a clonal lineage^22^. These studies could indicate the introduction and spread of the disease in Florida may be attributed solely to the asexual spores – the conidia. However the potential for long and short distance spread due to personnel and equipment moving inoculum must be considered. At this time, there is no evidence in the literature that the inoculum can be spread on clothing, equipment and/or machinery.

Rapid turnover and replanting of citrus trees within Grove II allowed for the investigation into the relationship between tree age and disease, unlike Grove I where there was little to no replanting. In Grove II, while mature fruiting trees were the predominant trees with CBS positive fruit, newly fruiting resets accounted for 13% of the positives finds during the 2015–2016 season. Previous literature has suggested and demonstrated that CBS infected dead twigs serve as a sources of inoculum^6, 21^ and this has been interpreted by some that only dead twigs can be colonized and serve as an inoculum source. Younger trees tend to have less woody branches and are less likely to contain dead twigs for *P*. *citricarpa* colonization. However, recent research had demonstrated that healthy green twigs are colonized by the fungus and once detached from the tree can produce viable inoculum within the first 45 days^27^.

The approach to sample size calculation was much different than formerly proposed by Sposito and colleagues^28^. Under the assumption that trees are evaluated at random, the type of spatial distribution of the CBS infection becomes irrelevant to estimating the proportion of CBS infected citrus trees. Of importance here is obtaining a sample size large enough to give an estimate within ±20% of the true population incidence of CBS. The sample sizes used in Groves I and II for each season of study proved more than adequate, especially as the incidence of CBS increased from year to year. The caveat to this is at very low or very high incidence of disease, simple random sampling may be inadequate to access the true disease incidence^29^, especially when individual trees are examined. However to overcome this inadequacy, quadrats can be established throughout the grove, with random quadrats as the sampling unit, this is the equivalent to cluster sampling^29^. In that case, all citrus trees within a randomly selected quadrat are examined and thus using a quadrat approach may be more efficient than randomly sampling individual trees in the grove.

Citrus black spot disease is clustered in the groves evaluated in this study in Florida, indicating short distance movement of the inoculum. The disease has the ability to progress rapidly under optimal environmental conditions of which southwest Florida exemplifies. Under heavy disease pressure, newly fruiting resets are more likely to have fruit exhibiting disease symptoms. Further understanding of the movement of inoculum in the grove and inoculum sources are required to devise better management systems to reduce spread of this disease.

## Methods

### Data collection

The incidence of trees with fruit exhibiting citrus black spot lesions was assessed in two commercial citrus groves in Florida between 2013 and 2016. In Florida, citrus is grown with irrigation ditches (swale) or a road (drivable surface) between alternating rows – row-ditch-row-road-row-ditch. Preliminary grove maps were prepared to assess the planting block. Next, fruits were evaluated between November and April of each citrus season following color break for the presence of hard spots. The hard spot lesion was chosen due to its ease of recognition and distinctiveness as a symptom of CBS. The exposed surfaces of fruits on each tree were observed for hard spots while walking along the swale and road^19^. If a single fruit on a tree was positive for blackspot, a thorough search of neighboring trees was carried out. The location of each tree with symptomatic fruit was plotted on their respective grove maps. Grove characteristics are given in Table 4.

**Table 4:** Characteristics of Valencia orange groves surveyed in Florida for citrus black spot, caused by *Phyllosticta citricarpa*.

Citrus Season	Grove	Cultivar/rootstock combination	Number of trees^1^	Tree spacing (m)	Percent positive^2^
2013–2014	I	Valencia/Swingle	2140	6.77 × 3.71	2.66
	II	Valencia/Swingle	4098	6.70 × 3.36	6.95
2014–2015	I	Valencia/Swingle	2140	6.77 × 3.71	3.50
	II	Valencia/Swingle	4365	6.70 × 3.36	17.41
2015–2016	I	Valencia/Swingle	2087	6.77 × 3.71	9.30
	II	Valencia/Swingle	4339	6.70 × 3.36	26.57

^1^Number of trees, this includes fruit bearing trees and resets that have not reached the fruit bearing stage. From year to year the number of trees may change due to tree removal and planting of resets.

^2^Percent positive reflects the number of trees with CBS positive fruit as a proportion of all trees in the planting including non-bearing resets.

### Weather Data

Groves are located in and around the Immokalee, FL area. Grove I (26°32′55.7″N 81°28′54.2″W) and Grove II (26°21′58.5″N 81°24′29.5″W) are approximately 10.46 km NNW and 11.08 km SSE of the Florida Automated Weather Network’s (FAWN) Immokalee station (26°27′43.5″N 81°26′25.9″W). The FAWN Immokalee station was used to gather 15 minute data on soil temperature, air temperature at 0.6, 2 and 10 m; relative humidity (RH) at 2 m (%), dew point at 2 m, rainfall (mm), wind speed at 10 m and solar radiation between January 1, 2010 and January 1, 2016. A 24-hr period was defined as starting at 12:00 am extending to 11:59 pm the following night. A rainy day was defined as a day when the measured rainfall was greater-than or equal to 0.25 mm within a 24 h period. A wet canopy (leaves and fruit) was defined as a day when the RH was equal to and greater than 90% for at least 8 consecutive hours^30^. Data on fruit set was extrapolated and estimated from data on flower bloom on the Flower Bud Induction Overview and Advisory web page (http://www.crec.ifas.ufl.edu/extension/flowerbud/index.shtml) and from the Citrus Flowering Monitor model at http://disc.ifas.ufl.edu/bloom. Search parameters were set to: Weather Station: Immokalee, Cultivar: Valencia, Expected Yield: Average/Low, Tree age: 4 or more years, Soil type: Deep sands and Date: according to year (2010–2015). Average duration of bloom in individual groves has been estimated at 12 to 20 days^31^. Bloom does not occur simultaneously, as such, estimated dates assumed to contain blooms were set to 9-days prior and –post bloom dates. Fruit set in ‘Valencia’ oranges is reliant on pollination. The number of days from pollination to fruit set was estimated as 14 days, based on work done by Mesejo and colleagues^32^. These data were used to estimate periods when conditions for infection were likely in the study groves. Fruit are susceptible to infection beginning at fruit set and extending up to 5 months following fruit set. From these data, three separate categories of potential for infection were defined:

IN1 = RH ≥ 90% for 8 hr + Total daily rainfall ≥0.25 mm + Temperature between 24 and 40 °C + susceptible fruit (fruit set to 5 months post fruit set).

IN2 = RH ≥ 90% for 8 hr + Temperature between 24 and 40 °C + susceptible fruit (fruit set to 5 months post fruit set).

IN3 = Total daily rainfall ≥0.25 mm + Temperature between 24 and 40 °C + susceptible fruit (fruit set to 5 months post fruit set).

### Spatial and Statistical analysis

The spatial pattern of trees with CBS positive fruit in each grove was analyzed using PROC SPP (SAS v9.4, SAS Institute Inc., Cary, NC). Briefly, a density-based approach was used to describe the first-order spatial properties of CBS in the mapped groves for each citrus season. Statistics based on nearest-neighbor distance and pairwise distances between points were used to test the hypothesis of complete spatial randomness (CSR) of disease (citrus black spot), that is, whether the diseased trees were clustered or not, in the study area of Grove I and Grove II.

Three nearest-neighbor functions, F, G and J, were used to assess clustering or regularity of trees with CBS positive fruit in each grove. These functions compare the empirical distribution function (EDF) calculated from the observed disease in the grove to the expected EDF for a pattern of CSR, in this case a homogenous Poisson process that has a first-order intensity λ, using a Monte Carlo simulation approach. The first-order intensity is computed from the number of trees with CBS positive fruit within the study area. The F-function is also known as the empty-space function, that is, it quantifies spatial interaction based on an event (tree with CBS positive fruit) proximity to voids. For distance r, when values of F_CBS_(r) < F_CSR_(r), and fall outside of the Monte Carlo envelopes, then the spatial pattern of trees with CBS positive fruit is statistically significantly clustered. The G-function quantifies spatial interactions based on event to nearest event distances, that is, from a tree with CBS positive fruit to the nearest trees with CBS positive fruit. In the case of the G-function, for distance r, when values of G_CBS_(r) > G_CSR_(r), and falls outside of the Monte Carlo envelopes, then the spatial pattern of trees with CBS positive fruit is statistically significantly clustered. Finally, the J-function compares the F-function and G-function and is written as:

1 $$J(r)=\,\frac{1-G(r)}{1-F(r)}{\rm{for}}\,{\rm{distancer}}\,r\ge 0\,{\rm{and}}\,F(r)\ne 1$$

A homogenous Poisson process has a theoretical value of 1, and clustering is indicated when J_CBS_(r) < 1.

Second-order characteristics, that is, pairwise distances between diseased trees were used to assess clustering versus regularity. Ripley’s K function, Besag’s L function and the pair correlation function were used to assess spatial patterns of citrus black spot in the study area of Grove I and Grove II. Ripley’s K function quantifies interaction in spatial point patterns by assessing the number of trees with CBS positive fruit within a radius, r, around each diseased tree. For Ripley’s K function, when values of K_CBS_(r) > K_P_(r) = πr^2^, for a homogeneous Poisson process within a distance of r from an arbitrary diseased tree, then the spatial pattern of CBS is clustered. In the case of Besag’s L function, which is a transformation of the K function, when values of L_CBS_(r) > L_P_(r), and falls outside of the Monte Carlo envelopes, then the spatial pattern of CBS is statistically significantly clustered. Besag’s L function was utilized for ease of interpretation as it has the properties of stabilizing the variance of the K-function and is relatively constant under CSR. Finally, the pair correlation function for a homogenous Poisson process has a theoretical value of 1, and clustering is indicated when *g*_CBS_(r) > 1 at distance r. Additionally, because all distance functions are influenced by edge effects, border edge correction is accounted for in the F, K and G functions^33^. All test were considered statistically significant at p < 0.05.

### Tree Age and Citrus Black Spot

During the 2013–2014 citrus season, at the beginning of the study, trees were either classified as resets (trees that were newly planted but had not begun to fruit) and fruiting trees – trees which contained citrus fruit at the time of the survey. In the 2014–2015 and 2015–2016 citrus seasons trees were further categorized into five groups based on approximate age and fruiting status. Tree were classified as indicated: 4 = trees > 4 years in the grove, fruiting during the 2013–2014 citrus season at the beginning of the study; 3 = fruiting resets, resets fruiting for the first time in the 2014–2015 citrus season (2–4 years); 2 = Resets, not yet fruiting (1.5–2 years); 1 = new resets and newly planted resets (1–1.5 years). Statistics based on nearest-neighbor distance and pairwise distances between points were used to test the hypothesis of complete spatial randomness (CSR) for resets bearing fruit for the first time in Grove II during the 2015–2016 citrus season. Additionally, CSR for fruiting resets with citrus black spot positive fruit during the 2015–2016 citrus season was analysed using PROC SPP (SAS v9.4, SAS Institute Inc., Cary, NC). The nearest-neighbor functions, F, G and J, and the second-order characteristics, Ripley’s K, Besag’s L and G functions were used to assess spatial patterns as previously described above. The distribution of trees (Category 3 and 4) among trees with CBS positive fruit were calculated and measures of association determined using the χ^2^-statistic (PROC FREQ; SAS v9.4, SAS Institute Inc., Cary, NC). All tests were considered statistically significant at p < 0.05.

### Sample Size Estimation

Data on the observed proportion of CBS positive events, P, either individual trees or quadrats with varying number of trees (2 × 2 to10 × 10; Table 3) from Grove I and II for each citrus season and overall incidence were used to generate estimates of sample size. Sample size estimation was made in order to answer two fundamental questions. (1) How many trees or quadrats need to be surveyed in order to accurately predict the incidence of disease within a FL grove with a relative precision of 20% and (2) how many citrus trees have to be surveyed in order to find fruit symptoms of the disease with 95% probability ? For both approaches the following assumptions were made:

(i) Sampling is carried out after color break when symptoms of citrus black spot are evident on the fruit.
(ii) Sampling of trees or quadrats is random without replacement, i.e. quadrats or individual trees within the grove are sampled randomly and once sampled will not be reassessed for disease within a single citrus season.

Given that sampling is completely random and without replacement, the number of infected sampling units, *m*, in a random sample of *n* infected sampling units has a hypergeometric distribution. Hence, based on large-sample theory for the sample proportion, the number of citrus trees or quadrats (sample size), *n*, needed to estimate disease incidence within a grove with (1−α)100% confidence and a relative precision of *ε* (%) is given by

2 $$n=\frac{N}{(1+{(\frac{\varepsilon p}{{z}_{1-\frac{\alpha }{2}}})}^{2}\frac{N-1}{p(1-p)})}$$

where *N* is the total number of citrus trees in the grove, *p* is the incidence of CBS and ${z}_{1-\frac{\alpha }{2}}$ is the standard normal distribution quantile associated with (1−α)100% confidence (e.g. *z* = 1.96 for α = 0.05).

The value used in eq. (2) for p should be based on prior knowledge or expert opinion as to the likely true incidence of disease in the grove. We calculated the required sample size needed for sampling individual trees and for sampling quadrats of size 2 × 2 to 10 × 10.

To estimate the number of trees that must be scouted in order to be (1−α)100% sure that the grove is infected, that is, number of trees assessed until the first CBS positive tree is observed we used the cumulative distribution function for the negative hypergeometric distribution

3 $$(1-{\rm{\alpha }})={\rm{\Pr }}(Y\le y)=\sum _{x=r}^{y}\frac{(\begin{array}{c}N-M\\ x-1\end{array})}{(\begin{array}{c}N\\ x-1\end{array})}\frac{M}{N-x+1}\,$$

where *N* is the total number of citrus trees in the grove and *M* is the number of trees with CBS. The number of infected trees to be observed before scouting is stopped and thus the grove is labelled as infected is set to *r* = 1 and the number of trees surveyed before disease is found is given by *y*, where *y* = (*r*, *r* + *1*, *r* + *2*, …, *r* + *N* − *M*).

The average number of trees per acre was calculated from the Florida Citrus Statistics 2014–2015 data, based on the total number of bearing orange, grapefruit and tangerine trees for that period, and the total estimated acreage. Calculations are based on the estimated trees per acre.
